# Cost-efficient production of in vitro *Rhizophagus irregularis*

**DOI:** 10.1007/s00572-017-0763-2

**Published:** 2017-02-16

**Authors:** Pawel Rosikiewicz, Jérémy Bonvin, Ian R. Sanders

**Affiliations:** Department of Ecology and Evolution, University of Lausanne, Biophore Building, 1015 Lausanne, Switzerland

**Keywords:** *Rhizophagus irregularis*, Arbuscular mycorrhizal fungi, Inoculum production, Root-organ culture

## Abstract

One of the bottlenecks in mycorrhiza research is that arbuscular mycorrhizal fungi (AMF) have to be cultivated with host plant roots. Some AMF species, such as *Rhizophagus irregularis*, can be grown in vitro on dual-compartment plates, where fungal material can be harvested from a fungus-only compartment. Plant roots often grow into this fungus compartment, and regular root trimming is required if the fungal material needs to be free of traces of plant material. Trimming also increases unwanted contamination by other microorganisms. We compared 22 different culture types and conditions to a widely used dual-compartment culture system that we refer to as the “standard system.” We found two modified culture systems that allowed high spore production and low rates of contamination. We then compared the two modified culture systems with the standard system in more detail. In the two modified culture systems versus the standard system, a comparable number of spores were produced per plate, the necessity for root trimming was reduced, and there was significantly diminished contamination in the fungal compartment. A cost analysis showed that both modified culture systems were more economic than the standard culture system for the production of the same number of non-contaminated spores. The two modified culture systems provide an economic alternative for the production of contaminant-free fungal material which is ideal for studies requiring AMF DNA or RNA for genetics, genomics, and transcriptomic studies or for studies requiring relatively large amounts of fungal material for greenhouse experiments.

**Electronic supplementary material:**

The online version of this article (doi:10.1007/s00572-017-0763-2) contains supplementary material, which is available to authorized users.

## Introduction

*Rhizophagus irregularis* is an arbuscular mycorrhizal fungus (AMF) that forms mutualistic symbioses with the roots of most land plants, improving their growth and resistance to environmental stress (Smith and Read 2010). Spores of *R. irregularis* can be produced in vitro for laboratory and greenhouse use or as a commercial inoculum that can be used to increase yields of commonly grown crop plants, such as potato (Wu et al. 2013; Hijri 2016), wheat (Al-Karaki et al. 2004), and cassava (Ceballos et al. 2013). For these reasons, the genomics, genetics, and transcriptomics of *R. irregularis* are being studied extensively in order to better understand the molecular genetics of plant-AMF interactions and also to produce a more effective inoculum (Sanders 2010).

One bottleneck in studying *R. irregularis* genetics and genomics is that the fungus has to be cultivated together with the host plant (Fortin et al. 2002). Thus, AMF spores can be contaminated with plant DNA and RNA. In order to produce spores of *R. irregularis* in vitro that are free of plant contamination, the fungus can be cultivated on dual-compartment plates (St-Arnaud et al. 1996). One compartment, called the plant compartment (Pc) contains carrot roots that have been transformed with *Agrobacterium rhizogenes*. The Pc is inoculated with *R. irregularis* that colonizes the carrot roots. Fungal hyphae can grow from the Pc to a second compartment, called the fungal compartment (Fc), where they proliferate and produce spores. This culture system can produce up to 34,000 spores in the fungal compartment (Fc) on a Petri plate. Spores can be collected from the Fc and used for experiments requiring the extraction of nucleic acids or to inoculate plants.

Even though sucrose is reduced in the Fc, thus inhibiting root growth, unfortunately, in this culture system, the plant roots can grow from the Pc to the Fc and can contaminate the medium with plant tissue. The solution to this problem is to trim the roots periodically and remove them manually from the Fc. *R. irregularis* is cultivated on a medium without antibiotics (Fortin et al. 2002), however, and root trimming greatly increases the risk of contamination with microorganisms. Moreover, root trimming is a labor-intensive task that limits the number of cultures that can be maintained simultaneously.

We aimed to improve the production of *R. irregularis* spores in the dual-compartment culture system that were free of contamination with microorganisms and plant material and that could be quickly produced without the necessity of root trimming. Ideally, this culture system should be cheap and easy to establish in basically equipped microbiology laboratories. For this purpose, we tried cultivating *R. irregularis* in 22 culture systems that were modifications of the method published by St-Arnaud et al. (1996). These modified culture systems were evaluated with respect to spore production and susceptibility of the Fc to contamination with either plant roots or unwanted microorganisms. We then selected the two most effective culture systems for evaluation in further detail in order to estimate the number of *R. irregularis* spores that could be produced with each and the cost of their production.

## Materials and methods

We established three separate experiments in order to find the most effective method to produce spores of *R. irregularis* free of contamination with plant roots and other microorganisms. Additionally, using data generated in experiments 2 and 3, we calculated the cost of producing one million spores with three different culture systems that were selected based upon the results of experiment 1.

### Biological material

We used *R. irregularis* isolate C3 in experiments 1, 2, and 3. Isolate C3 was obtained from an agricultural field in Tänikon, Switzerland (Anken et al. 2004) and has been maintained in vitro since 2000 (Koch et al. 2004). Additionally, in experiment 2, we also used *R. irregularis* isolate DAOM 197198 that was maintained under the same conditions as isolate C3. All culture systems in all the three experiments used the same stock of carrot roots that were transformed with *A. rhizogenes* as a host plant for the fungus (Bécard and Fortin 1988).

### Experiment 1—the evaluation of 22 different in vitro culture systems

Experiment 1 was established to quickly compare 22 different culture systems for their susceptibility to contamination and to quickly assess spore production. A list of all culture types and conditions evaluated in experiment 1 is given in Table 1.

**Table 1:** List of all culture types and conditions evaluated in experiment 1

Physically different types of culture system	Conditions				Contamination rate		Spore production		
	ID	Media type	Root trimming	Type of membrane or mesh	With microorganisms (%)	With plant roots in the Fc (%)	Number of plates categorized as		
							*H*	*I*	*L*
Standard culture system (Fig. 1a)	1	Ms	Yes	None	70	90	0	1	*9*
	2	Md	Yes	None	70	80	0	4	*6*
	3	Ms	No	None	20	100	0	4	*6*
	4	Md	No	None	10	100	1	*7*	2
Dual-compartment system with membrane placed on top of the Fc (Fig. 1b)	5	Ms	No	Cellophane	0	50	*4*	*4*	2
	6	Md	No	Cellophane	10	50	*7*	3	0
	7	Ms	No	PVDF	10	40	*7*	2	1
	8	Md	No	PVDF	10	30	*8*	2	0
	9	Ms	No	Mesh	0	80	*6*	3	1
	10	Md	No	Mesh	30	70	*7*	1	2
Dual-compartment system with liquid medium in the Fc (Fig. 1c)	11	Ms	No	None	100	0	0	0	*10*
	12	Md	No	None	100	0	0	0	*10*
	13	Ms	No	Mesh	80	0	0	0	*10*
	14	Md	No	Mesh	100	0	0	0	*10*
Large plate system (Fig. 1d)	15	Ms	Yes	None	80	100	0	*5*	*5*
	16	Md	Yes	None	70	90	1	*6*	3
	17	Ms	No	None	10	100	0	*8*	2
	18	Md	No	None	0	100	2	*6*	2
Large plate system with liquid medium in the Fc (Fig. 1e)	19	Ms	No	None	90	0	0	0	*10*
	20	Md	No	None	70	20	0	0	*10*
	21	Ms	No	Mesh	100	10	0	0	*10*
	22	Md	No	Mesh	70	30	0	0	*10*

The plates that were established in each culture system were classified as producing high (*H*), intermediate (*I*), or low (*L*) numbers of spores in the fungal compartment. The modal category of spore production is set in italics

*ID* identification number, *Ms* standard M medium as described in Online resource resource 1, *Md* double M medium as described in Online resource resource 1

### The standard culture system

**Fig. 1:**
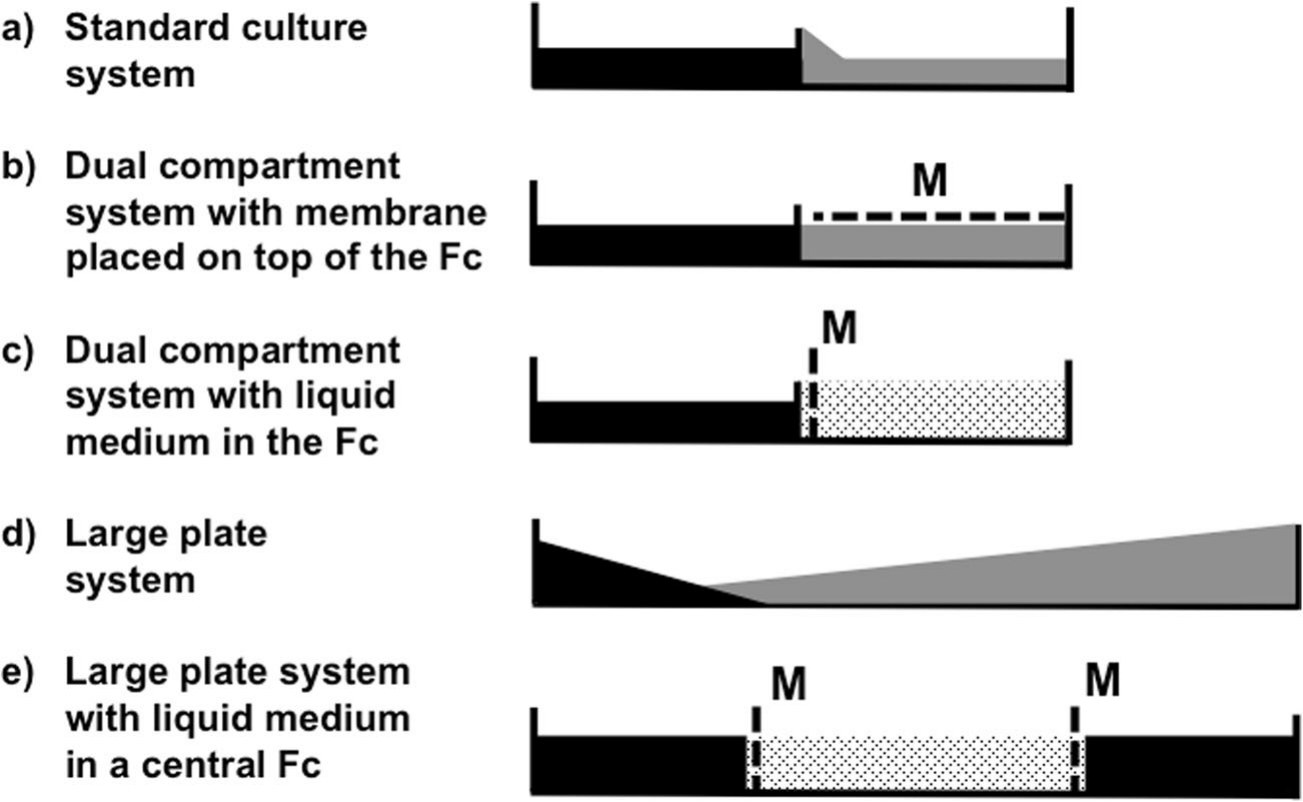
Five physically different types of culture system (**a**–**e**) that were evaluated in experiment 1. The plant compartment (Pc) is colored *black*. The fungal compartment (*Fc*) with a solid medium is colored *gray*, and the fungal compartment with a liquid medium is *dotted*. The membrane or nylon mesh (*M*) is depicted as a *dashed line*. Treatments with liquid medium in the Fc were established with nylon mesh (as shown in the figure) but also without nylon mesh (not shown in the figure)

We refer to the culture system that was established on a dual-compartment plate without a membrane or nylon mesh as the “standard culture system” (Fig. 1a). This is the culture method first described by St-Arnaud et al. (1996) and is one of the most suitable published methods available for the production of AMF spores that are free of plant roots.

### Culture systems compared with the standard system

We set up five physically different types of culture system (Table 1) of which the standard culture system was one. The cultures were established on dual-compartment Petri plates that were 9 cm in diameter (Fig. 1a–c) or on larger Petri plates, 14 cm in diameter (Fig. 1d, e). Each physically different type of culture system was established with two different compartments (the Fc and the Pc). Additionally, we used two different types of membranes or nylon mesh in order to prevent roots from entering into the Fc in three types of culture systems (Fig. 1b–e). They either were placed on top of the medium in the Fc (Fig. 1b) or placed vertically between both compartments (Fig. 1c, e). Moreover, in some culture systems, the Fc was filled with the liquid medium (Fig. 1c, e).

Each type of culture system was evaluated with two different media types: the standard M medium (Ms) and the double M medium (Md). Both media were modified versions of the M medium described by Bécard and Fortin (1988). Moreover, each medium was prepared in two different variants, which were used to separately fill in the Fc and the Pc on each Petri plate. The medium in the Fc lacked sucrose, and thus, suppressed root growth (Online resource resource 1). The liquid medium used in the Fc of some culture types had the same composition as the solid medium in the Fc of the other culture types, except that Phytagel was not added (Online resource resource 1). Additionally, 2 mm sterile glass beads were added to the liquid media in the Fc as a physical structure to support the growth of AMF hyphae. The media, glass beads, and membranes were sterilized at 121 °C for 15 min.

Carrot roots were trimmed manually in some cultures types, in order to reduce root growth in the Fc (Table 1). The roots were trimmed every 4 weeks. In other cultures, the roots were not trimmed. In the standard culture system, the frequency of root trimming was the only modification from the published protocol, as St-Arnaud et al. (1996) recommended weekly root trimming.

Two types of membranes or mesh were used. These were (i) a cellophane membrane (Sigma-Aldrich, Z377597-1PAK), (ii) a hydrophobic polyvinylidene fluoride membrane (PVDF, Millipore, HVHP09050), and (iii) a nylon mesh with 41 μm pore size (Millipore, NY4100010).

Ten Petri plates were established with each of the different culture types and conditions (shown in Table 1). Each plate was inoculated with carrot roots and a standardized amount of *R. irregularis* (isolate C3) in the Pc. To standardize the amount, a 2 × 2-cm piece of medium containing roots and *R. irregularis* was transferred to a new plate. For this, we only used plates where the roots covered the whole area of the Pc and the fungus appeared to have an even distribution across the whole of the Fc. The plates were wrapped with parafilm and stored inverted in an incubator in the dark at 25 °C for 3 months.

### Assessment of contamination and spore production

The plates were observed every 2 weeks under a stereomicroscope and checked for contamination with other microorganisms. In the case of plates that were contaminated, the number of AMF spores was estimated in the Fc and then these plates were discarded to reduce the risk of cross-contamination among the cultures.

We examined the cultures for the presence of plant roots in the Fc of each plate, 3 months after inoculation or at the time when the plates were discarded because of contamination with microorganisms.

**Fig. 2:**
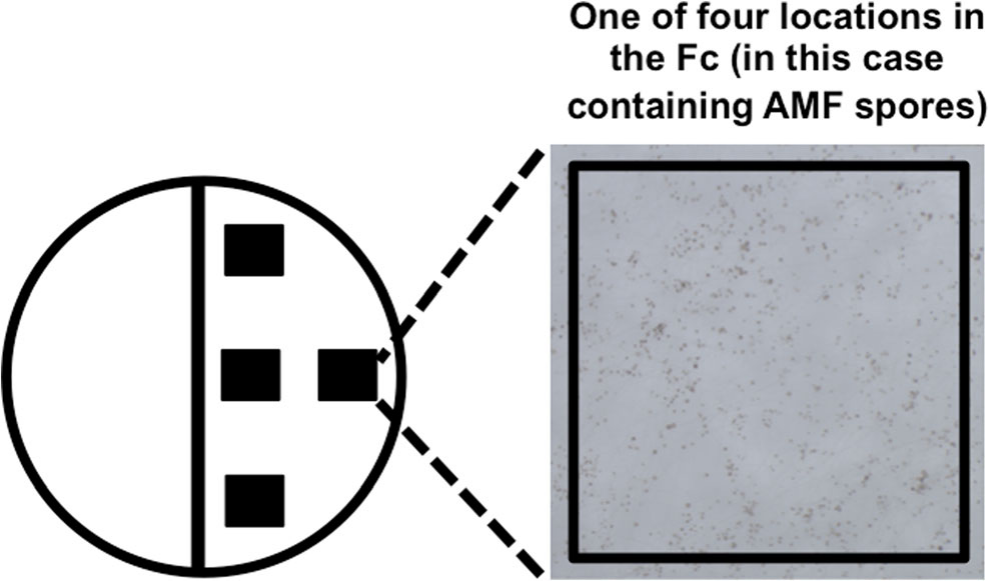
A culture system established on a dual-compartment plate with four stratified 1 × 1-cm locations shown as *black squares* in the Fc. These were used to estimate AMF spore production in experiments 1 and 2

The plates were classified in terms of AMF spore production in each type of culture system and in each condition. In order to do this, we examined the presence or absence of AMF spores in four stratified locations (1 × 1 cm) at the same position in the Fc on each Petri plate under a stereomicroscope (Fig. 2). Subsequently, plates with spores present in all four locations were classified as producing a high number of AMF spores. Petri plates with spores present in two or three locations were classified as producing an intermediate number of AMF spores. The plates with no spores or spores present in only one location were classified as producing a low number of spores.

### Experiment 2—spore production by two *R. irregularis* isolates in three different culture systems

Experiment 2 was established in order to estimate the number of AMF spores produced in the two culture systems that were identified in experiment 1 as the least susceptible to contamination in the Fc by microorganisms or plant roots. These were the dual-compartment systems with cellophane membrane and PVDF membrane placed on top of the solid medium in the Fc (Table 1; ID 6 and 8). We refer to these two culture systems as the “cellophane culture system” and the “PVDF culture system,” respectively. Additionally, in experiment 2, we estimated the number of AMF spores produced in plates that were established with the standard culture system (Table 1; ID 2).

We inoculated ten plates in each of these three culture systems with isolate C3 and another ten plates of each of these three culture systems with isolate DAOM 197198. Subsequently, the cultures were maintained for 3 months in the dark at 25 °C.

We used a stereomicroscope to count the number of spores in four different 1 × 1 cm locations on each plate in the Fc (Fig. 2). In order to estimate the total number of AMF spores produced on each plate, we calculated the mean number of spores per cubic meter of medium and this was multiplied by the medium volume in the Fc.

Data were transformed using a power transformation (0.3755) to give a normal distribution according to a Shapiro-Wilk normality test (*W* = 0.9848, *P* = 0.7204). There was no significant inequality in variance among treatments according to Bartlett’s test (*K* ^2^ = 9.6453, *P* = 0.08593). We tested for differences in mean number of *R. irregularis* spores in the Fc using a two-way ANOVA with culture system and *R. irregularis* isolate as the two factors. The effects of the culture systems on the number of spores were compared with a post hoc Tukey-Kramer honestly significant difference test (Tukey HSD). Probabilities of ≤0.05 were considered statistically significant. The statistical analyses were performed with the R programming language.

### Experiment 3—assessment of the number of plates contaminated with microorganisms or with plant roots in the Fc

Experiment 3 was set up in order to estimate how many plates that were established with the three different culture systems used in experiment 2 were lost to contamination with other microorganisms and contamination with plant roots in the Fc. For this purpose, we prepared 96 Petri plates of the standard culture system, 70 plates of the cellophane culture system, and 80 plates of the PVDF culture system. For each culture system, seven groups of plates were established (Online resource resource 2). Each group comprised from 6 to 28 Petri plates, depending on the availability of the starting material of isolate C3. The roots were trimmed every 4 weeks on plates established with the standard culture system and were never trimmed in the other two culture systems. All cultures were maintained in the dark at 25 °C for 3 months. Petri plates in each group were examined for microbial contamination and the presence or absence of plant roots in the Fc.

We calculated the percentage of plates contaminated with microorganisms and the percentage of plates with roots in the Fc in each group, giving seven values per culture system. The data were reported as mean percentage (±SE). The variance among treatments was homogenous (according to Levene’s test, *P* ≥ 0.05) and the data were normal (not truncated). No transformation of data was applied. The data were analyzed with one-way ANOVA to determine if there were significant differences in the percentage of plates contaminated with microorganisms among the culture systems. When the *F* ratio of the ANOVA was significant at *P* ≤ 0.05, we applied the Tukey HSD test. Because carrot roots were trimmed on all plates established with the standard culture system, we only tested for the difference in percentage of plates with roots in the Fc among plates established with the cellophane and PVDF culture systems using a Student’s *t* test. *P* values ≤0.05 were considered statistically significant. The statistical analyses were performed with the R programming language.

### Cost estimation

We used the data from experiments 2 and 3 to estimate the cost of producing a given number of AMF spores, in this case, one million, with standard, cellophane, and PVDF culture systems. The cost estimation was performed twice, under different assumptions. First, we estimated the cost of producing spores on plates that were free from contamination with microorganisms. We assumed that the plates contaminated with microorganisms were removed and that each plate with plant roots in the Fc was trimmed or the roots were cut and removed from the Fc before spore collection. Because small fragments of plant tissue can remain in the Fc after roots are trimmed, however, the spores potentially can be contaminated with plant DNA or RNA. Therefore, second, we estimated the cost of producing one million AMF spores on plates that are free of contamination with microorganisms and entirely without plant roots in the Fc. Furthermore, because all plates established with the standard culture system required root trimming, we only performed this estimation using the data for plates established with the cellophane and PVDF culture systems.

In order to estimate the number of plates free of contamination with microorganisms that were needed to produce one million spores, we used the data from experiment 2 (mean spore production per plate with each of the two different *R. irregularis* isolates; C3 and DAOM 197198) and the data from experiment 3 on loss of plates because of contamination with microorganisms. Furthermore, we estimated the cost of 1 unit (one plate) of each culture system so that we could estimate the total cost of production with standard, cellophane, and PVDF culture systems. This was calculated according to the cost of the laboratory materials in Switzerland at the time when this manuscript was submitted and did not include the cost of labor, because this differs greatly from country to country. In order to estimate the amount of labor, we calculated the number of person-hours needed to establish a given number of plates with each culture system and the number of person-hours required to trim the roots in the Fc of these plates. For this purpose, we assumed that one person could establish on average 20 plates/h with each culture system and that one person could trim the roots on 30 plates/h. Furthermore, we assumed that each plate established with the standard culture system that was contaminated with plant roots in the Fc required root trimming twice. Plates established with the cellophane and PVDF culture systems that were contaminated with plant roots in the Fc would have the roots cut and removed before spore extraction from the Fc. To calculate the number of plates that required root trimming with each culture system, we used the data from experiment 3 on the mean percentage of plates contaminated with plant roots in the Fc.

## Results

### Experiment 1

Cultures established with the standard culture system and with large plate systems in which root trimming was performed (Table 1; ID 1 and 2 and 15 and 16) were more susceptible to contamination with microorganisms than the cultures in the same culture systems in which root trimming was not performed (Table 1; ID 3 and 4 and 17 and 18). Furthermore, a great majority of plates (70 to 100% of plates) that were established with culture systems with liquid medium in the Fc (Table 1; ID 11–14 and 19–22) became contaminated with other microorganisms.

Nearly all plates established with the standard culture system (Table 1; ID 1–4) and with the large plate system (Table 1; ID 15–18) were contaminated with plant roots in the Fc. This occurred even though in some conditions the roots were manually cut and removed from the Fc (Table 1; ID 1 and 2 and 15 and 16). Additionally, we often found small fragments of plant material that remained in the medium and potentially could contaminate the spores. The carrot roots rarely grew from the Pc to the Fc in culture systems with liquid medium in the Fc. However, most of these cultures were contaminated with microorganisms or they did not produce spores in the Fc (Table 1; ID 11–14 and 19–22). The plates of the cellophane and PVDF culture systems (Table 1; ID 5–8) were more resistant to contamination with plant roots in the Fc than the plates established with any of the other culture systems with solid medium in the Fc (Table 1; ID 1–4, 9, and 10 and 15–18).

The cultures prepared with the Md medium produced a greater number of spores in the Fc more often than the cultures prepared with the Ms medium (Table 1). It is also interesting to note that the cultures which were subjected to manual root trimming produced fewer spores in the Fc than the cultures prepared in the same way without root trimming (Table 1; comparison between ID 1 and 3, 2 and 4, 15 and 17, and 16 and 18).

Experiment 1 allowed us to select the two most efficient culture systems for producing *R. irregularis* spores free of plant and microbial contamination. These were the cellophane and PVDF culture systems, both prepared with the Md medium (Table 1; ID 6 and 8, respectively).

### Experiment 2

The mean number of spores in the Fc differed significantly among plates established with the three different culture systems (ANOVA: *F*_2, 54_ = 17.4191, *P* = 1.445e−6; Table 2). Both isolates of *R. irregularis* produced significantly more spores in the cellophane and PVDF culture systems than in the standard culture system (Table 2). The mean number of spores in the Fc was significantly different on plates inoculated with the two different *R. irregularis* isolates (*F*_1, 54_ = 4.62, *P* = 0.036), with DAOM producing more spores than C3 in both modified culture systems (Table 2). There was no significant interaction between the culture system type and the AMF isolate.

**Table 2:** The mean (±SE), maximum, and minimum number of spores produced by two isolates of *Rhizophagus rhizophagus* (C3 and DAOM 197198) in the Fc on plates established with three different culture systems in experiment 2

*R. irregularis* isolate	Number of spores produced in three different culture systems		
	Standard	Cellophane	PVDF
C3			
Mean	4933 ± 2125	16,168 ± 2986	15,919 ± 3123
Maximum	17,348	22,713	33,248
Minimum	36	10,657	5633
DAOM 197198			
Mean	2295 ± 1924	24,658 ± 3435	28,294 ± 2893
Maximum	8931	49,155	61,395
Minimum	37	5682	10,987

### Experiment 3

As expected from the results of experiment 1, significantly fewer plates were contaminated with microorganisms when using the cellophane and PVDF culture systems (*F* ratio, *F*_2, 18_ = 4.67, *P* = 0.0034; Table 3). All plates established with the standard culture system were classified as producing spores that were potentially contaminated with plant roots, because small fragments of plant tissue often remained in the medium after the root trimming (Table 3). Approximately half of the plates of the PVDF and cellophane culture systems produced spores that were free of contamination with plant roots in the Fc. There was no significant difference in the mean percentage of plates with roots in the Fc in cellophane and PVDF culture systems (Student’s *t* test, *P* ≤ 0.05; Table 3). Additionally, we observed that the plates of the PVDF culture system were more resistant to drying than the plates of the standard and cellophane culture systems.

**Table 3:** The mean (±SE) percentage of plates established with the standard, cellophane, and PVDF culture systems that produced AMF spores free of contamination with microorganisms or that produced AMF spores free of contamination with plant roots and microorganisms

Plates that produced AMF spores free from contamination with:	Culture system		
	Standard	Cellophane	PVDF
Microorganisms	78.3 ± 7.1% (a)	99.6 ± 1.3% (b)	97.1 ± 1.7% (b)
Plant roots and microorganisms	None of plates	51.4 ± 2.4% (a)	57.1 ± 4.8% (a)

Means followed by the same lowercase letters in parentheses in the upper row do not differ significantly according to a Tukey HSD test (*P* ≤ 0.05). Means followed by the same lowercase letters in parentheses in the lower row do not differ significantly according to a Student’s *t* test (*P* ≤ 0.05)

### Cost estimation

#### Production of spores on plates free from contamination with microorganisms

Fewer plates were required to produce one million AMF spores using the cellophane and PVDF culture systems than using the standard culture method (Table 4 (on plates free of contamination with microorganisms)). Additionally, many plates established with the standard culture system were lost because of contamination with microorganisms. In contrast, almost no plates of the cellophane and PVDF culture systems were contaminated (Table 4 (on plates free of contamination with microorganisms)). Furthermore, the total number of plates needed to produce one million spores in each culture system greatly depended on the identity of the isolate of *R. irregularis*, especially with the standard culture system. All plates established with the standard culture system that were not contaminated with microorganisms, required manual root trimming. In comparison, less than half of the plates of the cellophane and PVDF culture systems required root trimming (Table 4 (on plates free of contamination with microorganisms)).

**Table 4:** Estimated cost of producing one million AMF spores on plates free of contamination with microorganisms and free of contamination with microorganisms and plant roots

	Isolate C3			Isolate DAOM 197198		
	Standard	Cellophane	PVDF	Standard	Cellophane	PVDF
On plates free of contamination with microorganisms						
Number of plates						
Required in total	259	62	65	557	41	36
Which required root trimming	203	30	26	436	20	15
Contaminated with microorganisms	56	<1	2	121	<1	1
Estimated cost (US $)						
In total	51.7	18.6	71.2	111.3	12.2	40.1
Per unit (plate)	0.2	0.3	1.1	0.2	0.3	1.1
Number of person-hours (h)						
In total	26.5	4.5	4.5	57.0	3.0	2.5
Required to prepare all plates	13.0	3.5	3.5	28.0	2.0	2.0
Required to trim the roots	13.5	1.0	1.0	29.0	1.0	0.5
On plates free of contamination with microorganisms and plant roots						
Number of plates						
Required in total		120	110		79	62
Contaminated with microorganisms and plant roots in the Fc		58	47		38	27
Estimated cost (US $)						
In total		36.1	121.0		23.7	68.1
Per unit (plate)		0.3	1.1		0.3	1.1
Number of person-hours (h)						
Required to prepare all plates		6.0	5.5		4.0	3.5

All plant roots were cut and removed from the Fc on plates that required root trimming prior to spore extraction. The plates contaminated with microorganisms were not used for spore extraction. Estimated number of person-hours was rounded up to 0.5 h. Cost was estimated according to the price of materials in Switzerland and converted to US dollars

Each plate in the PVDF culture system was approximately four times more expensive than a plate prepared for each of the other two culture systems. The cost for one PVDF culture system was US$1.1 per plate. Whereas, the estimated cost of one plate of the cellophane culture system and the standard culture system were US$0.3 per plate and US$0.2 per plate, respectively (Table 4 (on plates free of contamination with microorganisms)). The cellophane culture system was cheaper than the other two culture systems for spore production. The PVDF culture system was cheaper than the standard culture system in producing spores of isolate DAOM 197198 and the most expensive in producing spores of isolate C3 (Table 4 (on plates free of contamination with microorganisms)).

The production of one million spores of *R. irregularis* with the standard culture system required a greater number (5 to 22 times more) of person-hours than the production of the same number of spores with the other two culture systems (Table 4 (on plates free of contamination with microorganisms)). Moreover, root trimming on plates established with the standard culture system required more time than the preparation of these plates. In contrast, cutting and removing the roots from the Fc on plates established with either the cellophane or PVDF culture system required a relatively short amount of time (0.5 to 1 h; Table 4 (on plates free of contamination with microorganisms)).

#### Production of spores on plates free from contamination with microorganisms and plant roots in the Fc

Nearly half of the plates established with the modified culture systems were contaminated with microorganisms and potentially contaminated with small fragments of plant tissue (Table 4 (on plates free of contamination with microorganisms and plant roots)). Despite this, fewer plates were required to produce clean material with the two modified culture systems than with the standard culture system (Table 4 (on plates free of contamination with microorganisms and free of contamination with microorganisms and plant roots)). Moreover, the cost of spore production on plates of the cellophane culture system, free from contamination with plants roots in the Fc, was lower than the cost of using the standard culture system (Table 4 (on plates free of contamination with microorganisms and free of contamination with microorganisms and plant roots)). Furthermore, for a given isolate, both the modified culture systems required a similar amount of time in order to prepare the required number of plates (Table 4 (on plates free of contamination with microorganisms and plant roots)). The required time was greatly reduced in comparison with the required time to produce spores with the standard culture system (Table 4 (on plates free of contamination with microorganisms)).

## Discussion

The cellophane and PVDF culture systems produced a maximum of 49,155 and 61,395 spores per plate, respectively, although the mean numbers were much lower. These are a comparable number of spores to that obtained from a plate in the standard culture system (St-Arnaud et al. 1996). We observed that root trimming appeared to have a negative effect on spore production, probably because the AMF hyphae were also cut and removed from the Fc together with the plant roots. Additionally, root trimming strongly increased the risk of contamination with microorganisms because each Petri plate had to be opened several times to perform the procedure. The growing medium does not contain antibiotics, which makes it susceptible to contamination. The level of contamination because of opening the plates for trimming, however, likely will differ from laboratory to laboratory. Despite that plant roots were not trimmed on plates with the cellophane and PVDF culture systems, more than half of them were free of contamination with plant roots in the Fc. Moreover, we demonstrated that a larger number of spores could be produced more efficiently in each of these two culture systems than in the standard culture system, for a lower cost of materials and with a greatly reduced amount of labor.

The greatest advantage of the cellophane and PVDF culture systems is that a large number of spores completely free of contamination with microorganisms and plant tissue can be produced with relatively low labor time/costs. For this reason, new spores extracted from plates without roots in the Fc can be used to study AMF genetics, genomics, and transcriptomics with molecular techniques that require pure, high-quality fungal DNA or RNA. For example, the PVDF culture system already has been used successfully to produce a material from which to extract DNA of 20 different isolates of *R. irregularis* (Wyss et al. 2016). Those DNA samples from different isolates of *R. irregularis* could also have been produced by the standard culturing method, but it would have required more plates. Additionally, plant roots would have had to be cut often and removed from the Fc of each plate and AMF DNA still might be contaminated with a small amount of the plant DNA. In contrast, the same number of *R. irregularis* spores could be obtained easily with the PVDF or cellophane culture system with fewer than half as many plates as for the standard system, without root trimming and with less labor and material costs.

Another modification of the standard culture system that can lead to a production of 65,000 spores on average per plate has been proposed (Douds 2002)*.* That culture system, however, requires 9 months of cultivation, manual root trimming, and periodic exchange of the entire medium in the Fc every 2 months. In contrast, the two modified culture systems that we investigated produced almost half this number of spores on average per plate in half of the time and without any additional manipulation. Moreover, root trimming has been shown to be a labor-intensive task that reduces the total production of spores. Thus, our aim was to eliminate this procedure by using a membrane or mesh. Additionally, the plates established with the cellophane and PVDF culture systems with plant roots in the Fc can be trimmed. Trimming can be done just before spore collection, however, and this reduces the risk of contamination.

Approximately half of the AMF spores produced on plates established with the cellophane and PVDF culture systems potentially were contaminated with small fragments of plant tissue and therefore, may not be useful for DNA or RNA extraction, depending on the intended experiment. Nevertheless, these spores are still suitable for inoculating plants. Furthermore, many applications in biology such as flow cytometry or extraction of high length DNA require multiple-protocol testing and method calibration. Thus, these spores can be used routinely for protocol development.

To be successful, the important requirements of an efficient culture system are its low cost and reduced labor. The cellophane culture system was the most cost-efficient method over the short term. The total cost of each culture system strongly depends on both the mean number of spores produced on each plate by each isolate and the number of contaminated plates. For this reason, the PVDF culture system also potentially could be cheaper than the standard culture system, depending on which isolate is cultivated. Additionally, we observed that the plates of the PVDF culture system were more resistant to drying than the plates of the two other culture systems. Because some *R. irregularis* isolates produce spores more slowly than other isolates (Koch et al. 2004), it can be efficient to produce and store some isolates in the PVDF culture system, even though more expensive. Finally, our calculations were made for one production run or batch of one million spores. A factor that was not considered in our calculations, however, was that PVDF membranes can be cleaned, autoclaved, and re-used, unlike cellophane. Thus, this method can be highly suitable in a laboratory that has to continually produce uncontaminated AMF spores.

## Electronic supplementary material

Online resource 1 (DOC 54 kb)

Online resource 2 (DOC 44 kb)
