# Supplementary material for: Cost-efficient production of in vitro Rhizophagus irregularis
Source: Mycorrhiza. 2017 Feb 16;27(5):477–86. doi: 10.1007/s00572-017-0763-2 (PMC5486606; doi:10.1007/s00572-017-0763-2)
Supplement: Supplementary file 1 — (DOC 54 kb) [file 572_2017_763_MOESM1_ESM.doc]

**Online Resource 1**

**The composition of standard M medium (Ms) and double M medium (Md).**

**Article title:** Cost-efficient production of *in vitro* *Rhizophagus irregularis*

**Journal:** Mycorrhiza

**Authors :** Pawel Rosikiewicz, Jérémy Bonvin, Ian R Sanders

**Affiliation:** Department of Ecology and Evolution, University of Lausanne, Biophore Building, 1015 Lausanne, Switzerland

**Corresponding author:** Ian R.. Sanders; Tel: +41 (0)21 692 42 61; Fax: +41 (0)21 692 42 65; Email: [ian.sanders@unil.ch](mailto:ian.sanders@unil.ch)

**Online Resource 1** The composition of standard M medium (Ms) and double M medium (Md). There are two different variants of Ms and Md that are used to separately fill in the plant compartment (Pc) and the fungal compartment (Fc)

|  | **Ms (mg*l-1)** | |  | **Md (mg*l-1)** | |
| --- | --- | --- | --- | --- | --- |
| **Component** | **Pc** | **Fc** |  | **Pc** | **Fc** |
|  |  |  |  |  |  |
| MgSO_4_ . 7H_2_O | 731 | 731 |  | 731 | 1462 |
| Na_2_SO_4_ . 10H_2_O | 453 | 453 |  | 453 | 906 |
| KNO_3_ | 80 | 80 |  | 80 | 160 |
| KCl | 65 | 65 |  | 65 | 130 |
| KH_2_PO_4_ . 2H_2_O | 4,8 | 4,8 |  | 4,8 | 9,6 |
| Ca(NO_3_)_2_ . 4H_2_O | 288 | 288 |  | 288 | 576 |
| Sucrose | 30000 | - |  | 45000 | - |
| NaFeEDTA | 8 | 8 |  | 8 | 16 |
| KI | 0,75 | 0,75 |  | 0,75 | 1,5 |
| MnCl_2_ . 4H_2_O | 6 | 6 |  | 6 | 12 |
| Zn_2_SO_4_ . 7H_2_O | 2,65 | 2,65 |  | 2,65 | 5,3 |
| H_3_BO_3_ | 1,5 | 1,5 |  | 1,5 | 3 |
| CuSO_4_ . 5H_2_O | 0,13 | 0,13 |  | 0,13 | 0,26 |
| Na_2_MoO_4_ . 2H_2_O | 0,0024 | 0,0024 |  | 0,0024 | 0,0048 |
| Glycine | 3 | 3 |  | 3 | 6 |
| Thiamine hydrochloride | 0,1 | 0,1 |  | 0,1 | 0,2 |
| Pyridoxine hydrochlorine | 0,1 | 0,1 |  | 0,1 | 0,2 |
| Nicotinic acid | 0,5 | 0,5 |  | 0,5 | 1 |
| Myo inositol | 50 | 50 |  | 50 | 100 |
| Phytagel (P8169, Sigma) | 35 | 35 |  | 35 | 35 |
