# Supplementary material for: Cost-efficient production of in vitro Rhizophagus irregularis
Source: Mycorrhiza. 2017 Feb 16;27(5):477–86. doi: 10.1007/s00572-017-0763-2 (PMC5486606; doi:10.1007/s00572-017-0763-2)
Supplement: Supplementary file 2 — (DOC 44 kb) [file 572_2017_763_MOESM2_ESM.doc]

**Online Resource 2**

**Number of plates of each type of culture system in each group in Experiment 3 and the number of plates contaminated with microorganisms or with plant roots in the Fc**

**Article title:** Cost-efficient production of *in vitro* *Rhizophagus irregularis*

**Journal:** Mycorrhiza

**Authors :** Pawel Rosikiewicz, Jérémy Bonvin, Ian R Sanders

**Affiliation:** Department of Ecology and Evolution, University of Lausanne, Biophore Building, 1015 Lausanne, Switzerland

**Corresponding author:** Ian R.. Sanders; Tel: +41 (0)21 692 42 61; Fax: +41 (0)21 692 42 65; Email: ian.sanders@unil.ch

**Online Resource 2** Number of plates of each type of culture system in each group in Experiment 3 and the number of plates contaminated with microorganisms or with plant roots in the Fc

| **Culture system** | **Groups of Petri plates that were inoculated independently with AMF** | **Number of Petri plates** | | |
| --- | --- | --- | --- | --- |
|  |  | **prepared** | **contaminated with microorganisms** | **contaminated with  plant roots in the Fc** |
|  |  |  |  |  |
| **Standard** | I | 9 | 1 | 9 |
|  | II | 6 | 1 | 6 |
|  | III | 6 | 4 | 6 |
|  | IV | 6 | 1 | 6 |
|  | V | 20 | 2 | 20 |
|  | VI | 28 | 6 | 28 |
|  | VII | 21 | 2 | 21 |
|  |  |  |  |  |
| **Cellophane** | I | 10 | 0 | 4 |
|  | II | 10 | 0 | 5 |
|  | III | 10 | 1 | 4 |
|  | IV | 10 | 0 | 5 |
|  | V | 10 | 0 | 5 |
|  | VI | 10 | 0 | 5 |
|  | VII | 10 | 0 | 6 |
|  |  |  |  |  |
| **PVDF** | I | 10 | 0 | 4 |
|  | II | 10 | 0 | 7 |
|  | III | 20 | 2 | 8 |
|  | IV | 10 | 0 | 5 |
|  | V | 10 | 0 | 3 |
|  | VI | 10 | 1 | 4 |
|  | VII | 10 | 0 | 3 |
|  |  |  |  |  |
